# P055: Roommates’ colonization rates for multiresistant bacteria in a tertiary care hospital

**DOI:** 10.1186/2047-2994-2-S1-P55

**Published:** 2013-06-20

**Authors:** MJ Torijano, F Grande, M Cantero, M Grande, V Nováková, P Rodríguez

**Affiliations:** 1 Preventive Medicine and Quality Management, General University Hospital Gregorio Marañón, Madrid, Spain

## Introduction

Multiresistant-bacteria infection control measures in hospitals may include screening of patients at high risk of colonization, patients who are admitted to high-risk wards, patients submitted to high-risk procedures, roommates of patients with colonized or infected patients and screening during outbreaks.

## Objectives

To study the rate of multiresistant bacteria colonization in roommates of patients infected or colonized by multiresistant microorganisms.

## Methods

Retrospective observational study of smears collected from roommates of multiresistant-bacteria positive patients between 2010 and 2012. Percentages and 95% confidence intervals (CI95%) were calculated.

## Results

Altogether, 3306 roommates samples were collected, out of which 172 were positive (rate 5,20% (CI95%: 4,43-5,97)). Rates for MRSA were 6,50% (CI95%:5,14-7,86), extended-spectrum beta-lactamase-producing bacteria 4,33%(CI95%:3,3-5,36), and *Acinetobacter baumanii* multiresistant 4,48% (CI95%: 1,54-7,42), Carbapenemase-producing bacteria 1,85% (CI95%: 0,22-6,53). Rates along the study period were: 2010, 4,56% (IC95%: 3,50-5,62), 2011 (CI95%: 5,21-8,52) and 2012 3,82% (CI95%: 2,58-5,10).

## Conclusion

The percentage of positive samples in roommates of patients colonized or infected by multiresistant bacteria was relatively low during the studied period. However, further studies are needed to compare colonization rates between these roommates and other hospitalised patients to study if colonization can be due to sharing room in hospitals or not.

## Disclosure of interest

None declared.

